# The Constrained Disorder Principle: Beyond Biological Allostasis

**DOI:** 10.3390/biology14040339

**Published:** 2025-03-25

**Authors:** Ofek Adar, Josef Daniel Shakargy, Yaron Ilan

**Affiliations:** 1 Faculty of Medicine, Hebrew University, Jerusalem 9112001, Israel; ofek.adar@mail.huji.ac.il (O.A.); jdshakargy@gmail.com (J.D.S.); 2 Department of Medicine, Hadassah Medical Center, Jerusalem 9112001, Israel

**Keywords:** constrained disorder, allostasis, allostatic load, randomness, artificial intelligence

## Abstract

The constrained disorder principle (CDP) defines complex biological systems based on inherent variability. Allostasis refers to the physiological processes that help maintain stability in response to changing environmental demands. Allostatic load describes the cumulative wear and tear on the body resulting from prolonged exposure to stress, and it has been suggested to mediate the relationship between stress and disease. This study presents the concepts of CDP and allostasis while discussing their similarities and differences. We reviewed the current literature on the potential benefits of introducing controlled doses of biological noise into interventions, which may enhance the effectiveness of therapies. The paper highlights the promising role of variability provided by a CDP-based second-generation artificial intelligence system in improving health outcomes.

## Simple Summary

The constrained disorder principle (CDP) explains complex biological systems based on their natural variability. Allostasis is the process that helps the body maintain stability when facing changing environmental demands. Allostatic load refers to the wear and tear on the body from long-term stress, and it may play a role in linking stress to disease. This study discusses the ideas of CDP and allostasis, highlighting their similarities and differences. We reviewed current research on the potential benefits of introducing controlled amounts of biological noise into treatments, which could improve therapy effectiveness. The paper emphasizes the positive impact of variability from a CDP-based second-generation artificial intelligence system in enhancing health outcomes.

## 1. Introduction

Biological noise is recognized as an essential factor in biology and a vital design element in setting up treatment regimens [1]. It can be defined as the substantial cell-to-cell variation observed in populations of genetically identical cells [2]. Biological noise can also be observed in tissues and whole organs. The constrained disorder principle (CDP) defines complex systems by their inherent variability [3,4,5]. Allostasis refers to alterations in physiological set points following predictable and unpredictable changes [6]. Both theories examine how biological system stability can evolve from variability and how order evolves from disorder [7].

This paper presents the two theories and discusses their similarities and differences. We highlight the concept of adding noise to intervention regimens to improve efficiency and outcomes in medicine.

### 1.1. The Constrained Disorder Principle

The constrained disorder principle (CDP) defines complex systems based on inherent variability [3]. Per the CDP, every natural system is characterized by noise constrained by dynamic boundaries, which determine the range of the noise in a system. The dynamicity of the boundaries enables systems to adapt to changing environments. The CDP is presented by the B = F formula, where B stands for borders and F for functionality. The formula implies that the borders define the CDP. The CDP differentiates living from non-living systems based on their degree of variability. Per this principle, living systems have more variability constrained by dynamic boundaries than non-living systems. Per the CDP, a system requires a certain degree of noise for proper functioning. When the degree of noise is too high, outside the borders, or too low, inside the borders, it can lead to diseased conditions [4,5]. The concept aligns with the idea of the edge of chaos [8]. The CDP provides a platform for improving the efficiency of biological systems.

### 1.2. Allostasis

Sterling and Eyer first proposed the term “allostasis”, observing that many human pathologies are caused by alterations in physiological set-points rather than simple failures of homeostasis [9]. As a result, they replaced the term “homeostasis”, which was the preferred term for most body regulatory processes [10]. In homeostasis, the body adapts to environmental changes to maintain a stable physiological state. It means maintaining internal variables within a given set point. In allostasis, the body responds to changes in its environment with numerous physiological responses, allowing it to adapt to stressors and maintain homeostasis. Depending on the reaction, hormone levels, heart rate, blood pressure, and immune function may change, among others. The brain regulates allostasis by monitoring the body’s internal and external environments and adjusting the physiological responses accordingly [7]. In contrast to the reactive, thermostat-like models of homeostasis, allostasis contends that biology is dynamic and that set points rarely remain constant [11,12].

Allostasis resolves an inherent ambiguity in the term “homeostasis” by distinguishing between the systems that are necessary for life (“homeostasis”) and those that maintain these systems in balance (“allostasis”) in response to changes in the environment [6,13].

The “steady state” concept is vague and does not differentiate between essential life systems and those that maintain them [14,15,16]. Allostasis involves multiple systems in the body, including the endocrine, immune, and nervous systems, and it operates on numerous levels, from cellular to whole organism. An allostatic state emerges due to the action of primary mediators, hormones, elements of the immune system, and neural responses. Allostasis implies adjusting critical internal variables among set points [17,18].

### 1.3. For the Allostatic Load Model, Is There Pre-Planning?

Unlike neural or paracrine signaling, hormones mediate allostasis through broad spatial and temporal reach. Primary allostatic mediators include hypothalamic–pituitary–adrenal (HPA) axis hormones, catecholamines, and cytokines. Per this concept, glucocorticoids function as environmental response coordinators [7]. Allostatic load (AL) refers to the physical and emotional strain that results from activating the body’s stress response mechanisms [19]. Stressful situations trigger the release of catecholamines and glucocorticoids that allow the body to mobilize energy for the “fight or flight” response [20]. The ability to cope with stress is influenced by genetics, development, experience, health habits, and environmental factors. Allostasis coordination depends on the brain’s evaluation of threats and the regulation of physiological responses. The brain evaluates the threat and executes the appropriate physiological response based on differences between subjects [21].

In allostasis, allostatic load, allostatic overload, and homeostasis are combined to explain behavior and physiology in day-to-day physiology—predictive environmental changes, such as seasons, and optional responses to disturbances [7]. AL and allostatic overload involve multiple mediators of adaptation that are interconnected in a non-linear network. Each mediator system produces biphasic effects and is regulated by other mediators, leading to the non-linear impacts upon organs [21].

At first, prolonged secretion of the stress hormones epinephrine, norepinephrine, and cortisol, antagonized by dehydroepiandrosterone, or DHEA, can falter in their ability to protect the distressed individual and instead begin to damage the brain and body [22]. Stress hormones and their antagonists and pro- and anti-inflammatory cytokines represent the AL biomarkers and mediators [23]. These molecules affect cellular activities that compromise the physiological integrity of allostatic mechanisms. Over time, subsidiary biological systems compensate for the over- and under-production of these mediators and shift their operating ranges to maintain abated chemical, tissue, and organ functions. The prodromal stage involves metabolic biomarkers, visceral fat depositing, cardiovascular biomarkers such as systolic and diastolic blood pressure, and immune biomarkers. The final stage of AL progression is allostatic overload, whereby the culmination of physiological dysregulations leads to disordered, diseased, and deceased endpoints [24].

The AL model proposes that by measuring the multi-systemic interactions among primary mediators and effects, in conjunction with sub-clinically relevant biomarkers, biomedical advances can be made in the detection of individuals at high risk of severe outcomes [25,26]. Physicians routinely incorporate many of these biomarkers already, except attention is primarily placed on values reaching clinically significant levels. By combining and integrating additional biomarkers, identifying pre-clinical values, and triangulating methods with other feasible measures, including psychosocial, genotypes, and phenotypes, more prediction of pathologies can be achieved, which may prevent stress-related diseases [19].

The term “allostatic load” is used in various ways [19,27,28,29,30]. Some models propose a threshold concept, focusing on the endocrine “decision” to cross a threshold [7]. In allostasis and reactive scope models, “overload” is defined by elevated mediator levels, leading to variously activated emergency life-history stages (ELHSs) and damage [31]. Crossing such a threshold varies based on context, season, gender, and other factors [28,32,33].

Per the CDP, a stress response involves an alteration of the range for the inherent noise. It implies changing the borders of the noise in a system [3]. The variability in the degree of secretion of hormones, cytokines, and other mediators is altered in response to stressful situations. It is reflected by an increase or a decrease in the degree of variability and the change in the secretion level. Variabilities are part of the expected behavior of genes, tissues, blood pressure, respiration, gait, and brain function [34,35,36,37,38,39,40,41,42,43,44,45,46,47,48,49,50,51,52,53,54,55,56,57]. The boundaries-dependent mechanism enables the body to react to internal and external perturbations.

An example is heart rate variability (HRV), the beat-to-beat variation [48,49]. During stress, the heart rate increases; however, the HRV decreases, and an opposite effect is noted during meditation and sleep when the degree of variability rises [58,59,60]. HRV is controlled by the balance between the sympathetic and parasympathetic autonomous nervous systems, which implies the boundaries on the degree of variability [61,62]. A significant difference between allostasis and CDP is that there is no plan per CDP. The regulatory mechanisms are inherent to the system, exemplified by the boundaries. Under the CDP, there is no high-level regulation or long-term planning. Existence and efficiency are inherent to systems [4,5,44].

### 1.4. Energy Usage: Are We Wasting or Saving Energy to Keep It Balanced?

Allostatic load can lead to specific pathologies associated with chronic stress, particularly chronic exposure to elevated glucocorticoids in humans and laboratory animals. As the body transforms from allostasis to allostatic load, energy costs associated with allostasis and stress compete with growth, maintenance, and repair [63]. AL is the price that the body may pay to adapt to an unfavorable or deleterious physiological condition [6]. A framework based on energetics (E) was developed for allostatic load based on the recurring notion of “cost”. AL is a function of *E_e_* (basal existence) + *E_i_* (routine activity) + *E_o_* (unpredictable perturbations) [6]. Levels of glucocorticoids are predicted to change in parallel with AL.

In vertebrates, hormonal cascades, such as the hypothalamic–pituitary–adrenal axis (HPA), are vital in responding to perturbations across taxa [64]. Glucocorticoids play a role in energy balance that determines the responses to energetic demands and the AL and influences subsequent physiology and behavior associated with coping. Glucocorticoids are likely regulated by perturbation resistance potential (PRP), an indicator of an individual’s proximity to the energetic crisis [7,65]. An AL model quantifies PRP as the difference between available resources and allostatic load energetic costs, such as daily routines, life history stages (breeding, migration, and molt), and environmental perturbations. Spikes in blood hormone levels may reflect gradual or abrupt PRP changes. Individual responses to PRP vary and are related to hormone-metabolizing enzymes, mineralocorticoid receptors, and other downstream factors in target tissues. PRP, however, is difficult to measure. Elevation in glucocorticoids can be considered an endocrine “decision.” Error management can assess responsiveness to signs of impending energetic crisis. Intra- and inter-individual variations in responsiveness can be explained from this perspective [7].

Allostatic overload can be classified into two types: Type I occurs when the AL exceeds the environment’s energetic resources, and Type II occurs when the AL remains high without exceeding the basal energy available [7]. In Type II, food is defined as Eg (food available in the environment), energy is defined as Ecr (endogenous energy), and access to Eg is based on predation risk, social status, and other variables—allostatic overload Type II results in chronically elevated glucocorticoids [7]. As a result of allostatic overload Type 1, the animal is in negative energy balance. An ELHS is triggered, redirecting physiology and behavior to individual survival. It often interrupts normal life history stages, depending on the intensity and duration of the perturbation and individual trade-off decisions [6,7,66]. Elevated glucocorticoid hormone secretion triggers the ELHS. Normal fluctuations in glucocorticoid levels, such as those associated with the time of day, season, or life stage, differ from abnormally high levels related to *E_o_*. The ELHS is activated when glucocorticoids consistently elevate above the seasonal norm [28,66,67]. When an animal experiences chronically high AL without being in a negative energy balance, it suffers from allostatic overload Type 2. There is no ELHS triggered in this case. Hypertension, insulin resistance, and others may be symptoms of these imbalances [7,12,68].

The concept of predictive homeostasis refers to the range of ordinary activity (scope) of a mediator (like glucocorticoids) that occurs in response to reasonably predictable events (such as circadian changes, reproduction, or migration), which are components of Ee and Eo [69,70]. In reactive homeostasis, circulating mediators are above predictive homeostasis until they become damaging (wear and tear)—for example, healthy animals experience reactive homeostasis in response to an unpredictable perturbation [70]. Essentially, homeostasis overload occurs when a mediator’s blood levels rise above the reactive homeostasis range. Therefore, a mediator’s reactive scope spans predictive and reactive homeostasis ranges. Hormone levels range from low enough to maintain homeostasis to high sufficient to cause harm [7,17,28].

An appropriate time interval to average energy expenditure is necessary for an AL since significant moment-to-moment changes in energetic quantities should not result in overload. In active periods, the activity often appears in irregular bursts [71]. Unlike total energy expenditure over a day or longer, these frequent fluctuations do not reflect resource demand [63]. Some level of averaging is required for energy expenditure to reflect the AL. According to the variability in the duration over which an animal can tolerate negative energy balance as part of its daily or seasonal routine, the most reasonable time interval for averaging depends on species, sex, and season [7,63,72,73].

The CDP proposes that regulating the degree of noise in a system is a method nature uses for coping with perturbations while saving energy [3,4,5,45,74]. The degree of fluctuations in hormone secretion is regulated within a range. The fluctuation range can increase or decrease in response to environmental changes, serving as an energy-conserving mechanism and enabling a relatively low energy expenditure [3,5,45]. Per the CDP, “keeping in balance” implies staying at the “edge of chaos”, which requires less energy than the AL, which suggests using energy for changing set points [75,76].

### 1.5. Disease: An Allostatic Overload or Getting Out of the Boundaries?

Chronic or repeated exposure to stressors disrupts the allostatic process, negatively impacting health [25]. From an individual’s survival and well-being standpoint, mediators associated with allostasis play a crucial role in conferring short-term protective effects [22]. When hormone secretion becomes dysregulated, or an individual experiences adverse life events, a sustained state of allostatic load may ensue, leading to potentially harmful effects over more prolonged periods [21]. A high allostatic load can lead to chronic inflammation and metabolic dysfunction. Balancing stress and rest is essential for optimal health and well-being [77].

While acute activation of the sympathetic–adrenal–medullary (SAM) and hypothalamic–pituitary–adrenal (HPA) axes can help the body adapt to stress, chronic over-activation of these systems can lead to a “domino effect” on interconnected biological systems that overcompensate and eventually collapse, leaving the organism vulnerable to stress-related diseases [78,79,80]. Brain changes associated with chronic stress and AL, such as synaptic and dendritic remodeling, suppressed neurogenesis, and structural atrophy/hypertrophy, diminish the body’s ability to process stressors cognitively and respond [81]. These changes contribute to pathophysiological allostatic states that reflect response patterns in which allostatic systems are overactivated and dysregulated [23].

Per the CDP, a diseased state evolves when the degree of the inherent variability of a system gets out of control. It means having a too-small degree of noise, incompatible with dealing with dynamic environments and stress, or a too-high degree outside the boundaries [5]. Getting the disorder back to its range can serve as a mechanism for controlling a diseased process. While allostasis views the fluctuations as abnormal and as a target for control, per the CDP, systems function at the edge of chaos. There is always a degree of noise that changes according to the changing circumstances. The system has no exhaustion as the noise is mandatory for the proper function. Exhaustion can appear only when there is insufficient or too much noise in a system [4,5,34,35,36,37,38,39,40,41,42,43,44,45,46].

### 1.6. Predicting Adverse Health Outcomes and Mortality Risk

By including allostatic load in medical assessments, it may be possible to understand better a range of symptoms commonly encountered in clinical practice and identify previously unknown distinctions between seemingly similar patients [82]. Stressful experiences, whether acute or chronic, can have long-term consequences on an individual’s health. While genetic factors play a role in sensitivity to stress, individual responses are primarily determined by a person’s perception of the situation and their general state of physical health, influenced by lifestyle choices [21]. A correlation between elevated levels of allostatic load and overload and unfavorable health outcomes was proposed [82]. Allostatic overload is observed in numerous chronic illnesses, such as hypertension, coronary heart disease, congestive heart failure, diabetes mellitus, musculoskeletal disorders, neurological disorders, cancer, and psychiatric disorders [82].

Studies have demonstrated a correlation between allostatic overload and adverse health outcomes among cardiac patients. In a study of patients receiving implantable cardioverter-defibrillators, the sole significant predictor of unfavorable outcomes after the procedure, such as complications and mortality, was the presence of allostatic overload before the intervention [83]. A study investigating allostatic overload and congestive heart failure demonstrated a significant association between allostatic overload and hyperglycemia, a risk factor [84]. Allostatic overload was associated with lower well-being and quality of life [85]. A meta-analysis of 17 individual studies found that high allostatic load is a potent and emerging modifiable risk factor for all-cause and cardiovascular disease mortality, with an increased risk of 22% and 31%, respectively [86].

Per the CDP, early signs for prediction can be generated by determining changes in the degrees of variabilities in a system. Changes in the degree of variability provide a means for assessing the systems’ correct functioning and are a method for early disease detection, prognosis prediction, and response to therapy. A CDP-based digital twin model comprising biological noise was proposed to improve the prediction and detection of early signs of disease [5,74].

### 1.7. Coping with Changes

An organism’s life cycle depends on its ability to cope with environmental changes [87]. Day length, light intensity, temperature, and food availability are environmental cues that can predict life history stages and homeostatic adjustments [88]. Adaptation is necessary for organisms to deal with unpredictable events. An organism may modify its behavior or physiology to mitigate the effects of environmental changes called stress [89]. There is considerable overlap in how organisms cope, which varies according to body condition, social status, age, and sex. Some people find an environmental situation “stressful”, while others do not [90]. Predictable changes can be energetically demanding in allostasis, but individuals can prepare by anticipating the onset of life stages rather than responding facultatively [69,91]. As a result of such shifts, the body is continually stressed by ups and downs in physiologic response and by elevated activity of physiologic systems, metabolic changes, and wear and tear on organs and tissues, which is the allostatic load [69].

The CDP does not look into the degree of predictability of events or perturbation. Systems are designed to function under noisy environments and adjust their border to contain the required noise [3,4,5,74]. Dynamic borders ensure proper function by allowing different noise levels within a system depending on circumstances. As a result of this “automatic” mechanism, which characterizes systems at all levels, systems can cope with changes. The CDP does not look at perturbations as continuous stress. Unpredictable events are not differentiated from predictable ones as they are not meant to be “prepared” beforehand. The CDP implies that molecules do not predict. The adaptability to changes manifests itself by comprising more or less noise in a system [4,74].

### 1.8. Intra and Inter-Individual Variation: Plasticity

Per allostasis, environmental changes are expected to lead to most types of variability occurring. An organism can learn to optimize its response to general and cue-specific environmental perturbations using developmental plasticity, habituation, and sensitization [92]. Plasticity can occur within or across generations and is an alternative to relying on instinctive responses shaped by evolution in a changing environment. Evolution likely shapes the extent to which such plasticity can occur in changing environments [92]. The presence of novel environments should initially cause generalized sensitization since they reflect unknown cue reliability [17]. However, the ability of animals to habituate to “stressors” demonstrates their ability to learn about cue reliability and reduce responsiveness accordingly. Habituation to novel stimuli is sometimes accompanied by the potentiation of response to other novelties, i.e., sensitization. Such adaptation to novelty is apparent, as is a protective response against repeated false positives [7,93].

Per the CDP, a certain degree of variability exists in all systems and is mandatory for proper function. The changes in the boundaries of the variability reflect the plasticity of a system by which it adapts to changes [3,4,5]. System variability is inherent to all levels, from DNA to tissues and whole organs, so omitting it is not a goal [34,35,36,37,38,39,40,41,42,43,44,45,46,47,48,49,50,51,52,53,54,55,56,57]. The CDP views interindividual differences similarly to intraindividual plasticity. Similar changes in an individual’s environment alter the variability borders differently in different subjects. A difference in the dynamicity of the borders between individuals explains this phenotypic difference [74]. Variation between individuals can result from the very different environments experienced by individuals [25,89,94,95]. It may also reflect phenotypic diversity in hereditary responsiveness. Behavioral phenotypes with distinct aggression and stress response profiles can be classified as “Hawks” or “Doves”, both advantageous and disadvantageous, maintaining both over time [96]. Epistatic effects between individuals may complicate the impact of social structure on genetically heritable phenotypes. To some extent, however, such phenotypes may be established by epigenetic mechanisms during development. Species may differ significantly in the extent to which such developmental programming occurs or endures [78,97].

### 1.9. The Benefits of Adding Biological Noise to Improve Health Outcomes

Biological noise refers to the inherent variability in biological systems that arises from the stochastic nature of biochemical reactions and physiological processes [38,39,40,41,42,43,44,45,46,47,48,49,50]. Recent studies have shown that adding controlled biological noise to systems could benefit health [98,99].

In the allostasis theory, biological noise can induce a state of allostasis, or dynamic stability, in which the system can better adapt to stressors and maintain homeostasis. Adding biological noise to induce allostasis and alleviate allostatic load was proposed to improve health outcomes [95,100]. One potential application of this concept is in developing personalized medicine strategies. Adding carefully calibrated amounts of biological noise to a patient’s physiological systems may enhance their ability to cope with stressors and improve their health outcomes. It could involve techniques such as stochastic resonance, which consists of adding a low noise level to a system to improve its signal-to-noise ratio and enhance its ability to detect weak signals [101]. Another potential application is in the field of mental health. Studies have suggested that exposure to controlled stress can help build resilience and enhance mental health outcomes [102]. Adding biological noise to mental health interventions, such as cognitive-behavioral therapy, stress inoculation training, or mindfulness meditation, may help to induce a state of allostasis and promote greater resilience in patients [102,103].

Both allostasis and the CDP look at the favorable effect of noise. However, per the CDP, noise is inherent to all systems at all levels. Noise is not something that needs to be added. The CDP implies that noise is inherent to systems, and changes in the noise level within a system indicate disease. A system’s adaptability consists of managing its noise; therefore, the CDP does not add noise. Increasing the noise level in low-noise systems can be achieved by widening the noise boundaries, while decreasing the noise level in high-noise systems can be achieved by tightening the boundaries [3,74]. Allostasis looks at adding noise to reach a new set point as part of the adaptability. In contrast, per the CDP, systems are always at the edge of chaos, and adaptability does not mean changing set points but changing the boundaries of the noise in response to perturbations. While noise in the allostasis theory involves energy consumption, per the CDP, the dynamic borders are a relatively energy-conserving mechanism that nature has for adaptability.

The CDP concept was suggested to overcome tolerance to drugs in patients with chronic diseases in whom a partial or complete loss of response to medications is common [104]. Different mechanisms are associated with the loss of response to therapies in patients with chronic diseases, and introducing noise into the treatment regimen can lead to regaining the effect. According to the CDP, noise is inherent to systems, and regular drug administration does not account for this intrinsic variability [105,106,107,108,109,110,111,112,113,114,115,116,117,118,119,120,121,122,123,124,125]. A second-generation artificial intelligence (AI) system was developed to introduce variability into therapeutic regimens to improve outcomes [105,106,107,108,109,110,111,112,113,114,115,116,117,118,119,120,121,122,123,124,126,127,128,129,130,131,132,133,134,135,136,137]. The platform enables tailoring personalized variability-based treatment regimens by introducing noise based on clinically meaningful outcome parameters [137]. The system improved clinical and laboratory measures in patients with chronic heart failure who developed diuretic resistance and reduced emergency room and hospital admissions associated with heart failure [138]. This system showed similar benefits in patients with multiple sclerosis and chronic pain by introducing variability into the treatment regimen [74]. The clinical data support the CDP by showing that regulated noise in therapies can improve outcomes.

Table 1 demonstrates some similarities and differences between CDP and allostasis.

### 1.10. How Homeostasis, Allostasis, and the CDP Define and Treat Hypertension

Hypertension is a chronic medical condition that affects millions of people worldwide. It is a significant risk factor for cardiovascular diseases and other health complications. Blood pressure variability is a normal physiological finding [139,140,141]. These findings dictate that the goal of blood pressure treatment should be to maintain blood pressure under specific values and control variability. Hypertension can provide a valuable illustration of how homeostasis, allostasis, and the CDP define a chronic disease state and respond.

Homeostasis, the body’s ability to maintain internal stability and balance, implies that in a healthy individual, homeostatic mechanisms regulate blood pressure within a narrow range. Hypertension can be viewed in this contest as a disruption of homeostasis and the body’s ability to regulate blood pressure effectively, indicating an imbalance in the physiological mechanisms responsible for maintaining stability [142]. Homeostasis would be keeping the blood pressure around the same levels throughout the day.

AL implies that blood pressure changes are embedded in the physiology by using catecholamines and glucocorticoids. However, when a threshold is crossed, and the load exceeds the physiological capabilities, permanent changes may occur to the physiological set-point and other systems suffering, such as endothelial damage due to excessive blood pressure as a pick or as a constant [143,144]. Understanding the homeostatic mechanisms involved in blood pressure regulation can guide treatment approaches for hypertension. Medications targeting specific components of these mechanisms, such as angiotensin-converting enzyme inhibitors or beta-blockers, are prescribed to restore homeostasis and reduce blood pressure to return the levels to normal values [145].

Allostasis defines hypertension as a consequence of the body’s attempt to adapt and achieve stability in the presence of ongoing stressors, highlighting the dysregulation of multiple systems involved in blood pressure regulation [146]. Allostasis suggests adjusting the parameters to the circumstances, e.g., when physically active. Per allostasis, the body’s adaptive response to maintain stability in the face of stressors, including psychological stress, triggers an allostatic response, leading to prolonged activation of stress-related physiological pathways. This chronic activation contributes to the development and progression of hypertension. The allostatic overload among hypertensive patients is associated with lower well-being and quality of life [85]. Recognizing the impact of chronic stress on hypertension suggests a need for a comprehensive treatment approach. Strategies to manage stress, such as relaxation techniques, exercise, and counseling, can be incorporated alongside pharmacological interventions to address the underlying allostatic load and reduce blood pressure [103]. The goal is to reach a new set point based on the situation.

The CDP provides an alternative view on the definition of hypertension by focusing on the dynamic balance between order and disorder in biological systems. In this context, adaptation to chronic or repeated exposure to stressors involves changes in the range of “noise” within dynamic boundaries. Hypertension arises when these noise boundaries fail to regulate blood pressure effectively, leading to persistently elevated or low noise levels outside the established limits. These boundaries define the acceptable range of noise as a mechanism for adaptation. When they malfunction, the noise levels can become excessively high or too low [3,4,5,74,104,135,136,137,147,148,149,150,151,152].

Applying the CDP to the treatment of hypertension highlights the significance of stability and adaptability. Treatment strategies should focus on restoring the disrupted balance of noise, whether the levels are too low or too high, by addressing the underlying causes of hypertension. It is essential to consider individual variations and constraints while maintaining a position at the “edge of chaos”. This concept suggests that noise levels can vary depending on different circumstances. It calls for introducing variability into medications, lifestyle modifications, and ongoing monitoring to optimize blood pressure control under different settings rather than applying therapies to reach a target level. There is no new set point but a continuing adaptation to dynamic perturbations. Including noise in intervention regimens, including drug therapies, is proposed to make them more physiologically appropriate, overcome tolerance, and improve clinical outcomes [4,5,150].

CDP-based artificial intelligence systems that alter chronic medications’ dosages and administration times within the approved range, known as the digital pill, have been shown to improve effectiveness, overcome tolerance, and reduce side effects [74,131,136,138,153].

**Figure 1:**
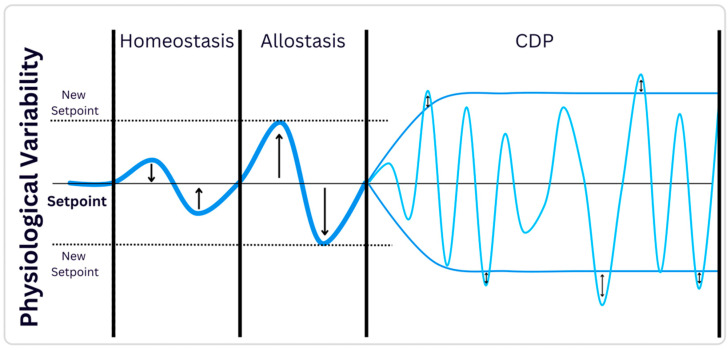
A schematic representation of homeostasis, allostasis, and the constrained disorder principle (CDP). All theories involve the response to environmental perturbations. The forces and energy push the system back to its set point in homeostasis. In allostasis, the forces set a new point based on the circumstances, demanding energy expenditure. Per the CDP, each system has an inherent disorder that is mandatory for its proper function. Adapting to changes does not involve predictability and is conducted by alterations in the dynamic borders, which regulate the level of noise in the system. In allostasis, each of the three set points represents a range of different values associated with noise, and these distributions may overlap. The set points can belong to the same or different but related systems. In contrast, the CDP does not have set points; instead, the boundaries for noise are dynamic and change based on the environment. The small arrows illustrate the variability of these noise boundaries, highlighting that the noise level within a system may adjust to adapt to environmental changes.

Figure 1 schematically illustrates the differences between the homeostasis, allostasis, and CDP theories.

## 2. Conclusions

Adapting to changes and perturbations is mandatory for the proper function of systems. Homeostasis, allostasis, and the CDP provide models for living under dynamic changes. Homeostasis is based on corrections by returning to the baseline set point. Allostasis is based on a change in the set points. Charles Darwin is often quoted as saying “It is not the strongest of the species that survives, neither is it the most intelligent that survives. It is the most adaptable to change” [154]. While it is debatable whether Darwin said it, the concept resonates with the CDP. The CDP views biological systems as having inherent variability and living at the edge of chaos, which is mandatory for proper function. Per the CDP, “set-points” are part of the dynamic boundaries that determine the noise degree based on the changing circumstances. The CDP does not provide molecules with a prediction ability. It is about changing the level of noise in response to perturbations. The CDP offers a platform that can be used to improve the function of biological systems by regulating the degree of noise in interventions in a personalized way.

## Figures and Tables

**Table 1:** Several differentiators between the constrained disorder principle (CDP) and allostasis.

	CDP	Allostasis
Noise	Noise is inherent to all systems, and its dynamic borders determine their adaptability.	Allostasis accounts for noise. Systems reach a set point for omitting the noise in the surroundings.
Adaptability	Biological systems adapt to perturbations by changing the range of the internal noise.	Adaptability is based on changing set points in response to perturbations.
Equilibrium	Systems function at the “edge of chaos” and always manifest a certain degree of noise, which is mandatory for proper functioning.	Systems reach new equilibrium positions by mechanisms involving multiple hormones and cytokines in response to environmental stress.
Planning ahead of time	Under the CDP, there is no high-level regulation or long-term planning. Existence and efficiency are inherent to systems.	Central control in the brain is associated with planning for a stressful situation and directing the response.
Energy expenditure	Regulating the degree of noise in a system is a method nature uses to cope with perturbations while saving energy. The CDP focuses on saving energy by regulating noise in the system, omitting resistance to changes, or using forces to change set points.	As the body transforms from allostasis to allostatic load, energy costs associated with allostasis and stress compete with growth, maintenance, and repair. Glucocorticoids play a role in energy balance, determining responses to energetic demands (allostatic load) and influencing subsequent physiology and behavior associated with coping.
Reaching stability	The body adapts to changes in its internal and external environments to maintain stable function, even if this means continuous instability.	The body can adapt to changes in its internal and external environment to maintain a stable physiological state.
Scope of influence	All body functions, from the DNA to whole organ levels, are subject to the process of regulating noise.	Depending on the reaction, hormone levels, heart rate, blood pressure, and immune function change.
Regulatory mechanisms	Regulation occurs at the cell, tissue, or organ levels in parallel to central regulation by the brain.	The brain regulates allostasis by constantly monitoring the body’s internal and external environment and adjusting physiological responses accordingly.
Disease states	Chronic or repeated exposure to stressors does not disrupt function, which is met by changes in noise ranges by the variable dynamic boundaries. Disease states are associated with boundary malfunctions and too much or too little noise in the system.	Chronic or repeated exposure to stressors disrupts the allostatic process, negatively impacting health.
Balance	The CDP does not aim to reach a balance; living at the edge of chaos is essential for proper function.	Balancing stress and rest is crucial for optimal health and well-being.
Differentiation of functional systems	The CDP does not distinguish between systems; all body systems are mandatory for function and react to perturbations.	Allostasis distinguishes between critical systems for life (“homeostasis”) and systems that maintain balance (“allostasis”) in response to environmental and life stage changes.
Equilibrium set points	The CDP implies that biology is dynamic, with no specific “final set point” for which the body aims. Systems function at the edge of chaos.	Allostasis contends that biology is dynamic, and set points rarely remain constant, contrary to reactive, thermostat-like homeostasis models. A new set point is the new equilibrium state.
Predicting adverse health outcomes	Variability is a measure of system functioning. It enables early disease detection, prognosis evaluation, and monitoring of therapeutic responses. Assessing a system’s variability level can yield early prediction indicators.	The allostatic load model proposes that measuring multi-systemic interactions among primary mediators and effects, along with sub-clinically relevant biomarkers representing secondary outcomes, can enhance the prediction of pathologies, aiding in the prevention of stress-related diseases.
Plasticity	Changes in variability boundaries represent the system’s plasticity and ability to adapt to changes.	Plasticity can occur within or across generations, allowing organisms to optimize their responses to general and cue-specific environmental perturbations.
Impacts of biological noise	Widening or tightening variability borders in low or high biological noise systems may improve medication response by overcoming drug tolerance.	Controlled exposure to biological noise may enhance a patient’s ability to cope with various stressors, induce allostasis, and improve overall health outcomes.
Adding noise to improve outcome	Noise is inherent to all systems at all levels. The widening or tightening of its boundaries serves as an adaptation mechanism to regulate the amount of noise in a system to improve its function. If the noise is too high, it implies a need for its constraints; if it is too low, widening the boundaries enables it to increase. The goal of regulating noise is not to reach a new set point but to improve functioning at the edge of chaos in an energy-conserving way.	Adding noise may require systems to spend energy to reach a new set point and equilibrium state.
Timeframe	Highlights the dynamic nature of noise and adaptation in real time without considering specific timeframes	It encompasses acute responses to immediate stress challenges and chronic adaptations over extended periods.
Impact of chronic stress	Chronic stress is accommodated by adjusting noise ranges within dynamic boundaries.	Chronic stress disrupts allostasis processes, leading to maladaptive changes and allostatic load and overload.

## Data Availability

No data were generated in the present study.

## Abbreviations

CDP	Constrained disorder principle
HPA	Hypothalamic–pituitary–adrenal
AL	Allostatic load
DHEA	Dehydroepiandrosterone
ELHS	Emergency life history stage
HRV	Heart rate variability
*E_e_*	Minimum energy level required to maintain homeostasis
*E_i_*	Energy needed to make predictable changes under ideal conditions to adjust to environmental changes
*E_o_*	Energy cost for unpredictable perturbation
Eg	Energy in the environment
Ecr	Endogenous energy
PRP	Perturbation resistance potential
SAM	Sympatheticadrenalmedullary
DNA	Deoxyribonucleic acid
AI	Artificial intelligence
